# Predicting the Functional Outcome of Adult Patients with Status Epilepticus

**DOI:** 10.3390/jcm8070992

**Published:** 2019-07-08

**Authors:** Chih-Hsiang Lin, Chen-Jui Ho, Yan-Ting Lu, Fu-Yuan Shih, Yao-Chung Chuang, Meng-Han Tsai

**Affiliations:** 1 Department of Neurology, Kaohsiung Chang Gung Memorial Hospital, College of Medicine, Chang Gung University, Kaohsiung City 83301, Taiwan; 2 Department of Neurosurgery, Kaohsiung Chang Gung Memorial Hospital, College of Medicine, Chang Gung University, Kaohsiung City 83301, Taiwan; 3 Department of Biological Science, National Sun Yet-Sen University, Kaohsiung City 80424, Taiwan; 4 School of Medicine, College of Medicine, Chang Gung University, Taoyuan City 33302, Taiwan

**Keywords:** status epilepticus, functional outcome, STESS, EMSE, END-IT

## Abstract

Patients that survive status epilepticus (SE) may suffer from neurological and cognitive deficits that cause severe disabilities. An effective scoring system for functional outcome prediction may help the clinician in making treatment decisions for SE patients. Three scoring systems, namely the Status Epilepticus Severity Score (STESS), the Epidemiology-Based Mortality Score in Status Epilepticus (EMSE), and the Encephalitis-Nonconvulsive Status Epilepticus-Diazepam Resistance-Image Abnormalities-Tracheal Intubation (END-IT), have been developed in the past decade to predict the outcomes of patients with SE. Our study aimed at evaluating the effectiveness of these scores in predicting the function outcomes both at and after discharge in SE patients. We retrospectively reviewed the clinical data of 55 patients admitted to our neurological intensive care unit between January 2017 and December 2017. The clinical outcomes at discharge and at last follow-up were graded using the modified Rankin Scale. Our research indicated that STESS was the most sensitive and EMSE was the most specific predictive scoring method for SE outcome prediction. On the other hand, END-IT predicted functional outcomes in SE patients poorly. We concluded that STESS and EMSE can accurately predict the functional outcomes in SE patients both at discharge and the follow-up period.

## 1. Introduction

Status epilepticus (SE) is a neurological emergency with a mortality rate ranging between 7.6% and 39% [1]. Even though several consensus guidelines on SE treatment have been published [2], the prognostic outcome remains suboptimal in SE patients. The aggressiveness of SE management generally depends on the predicted outcome at presentation [3]. For patients with a predicted poor prognostic outcome, vigorous monitoring and aggressive seizure treatment can be performed in order to avoid under-detection or under-treatment of SE. However, for patients with a predicted good prognostic outcome, a less aggressive therapeutic approach should be adopted in order to avoid potentially harmful over-treatment. Moreover, this would reduce unnecessary expenditure on medical resources. Thus, in order for neurologists to evaluate risks and accurately predict prognostic outcomes in SE patients, an informative and reliable SE outcome prediction method is required.

During the past decade, three scoring systems, namely the Status Epilepticus Severity Score (STESS) [4], the Epidemiology-Based Mortality Score in Status Epilepticus (EMSE) [5], and the Encephalitis-Nonconvulsive Status Epilepticus-Diazepam Resistance-Image Abnormalities-Tracheal Intubation (END-IT) [6], have been developed to predict SE outcomes. The STESS [4] predicts patient outcomes based on clinical information obtained pre-treatment and represents a relatively rapid and straightforward analysis that can be conducted during the initial presentation. On the other hand, for the EMSE and END-IT outcome-prediction methods, additional data are required. These include the pattern of the worst electroencephalography (EEG), SE etiology, whether or not the SE is nonconvulsive, and the analysis of brain image [5,6]. However, this information can in general only be acquired after initial presentation and may change during the course of hospitalization.

Recently, a study investigated the accuracy of STESS, modified STESS, EMSE-EAL (EMSE including only etiology, age, and level of consciousness), and END-IT in outcome-prediction for in-hospital mortality. The results indicated a low specificity for STESS, balanced sensitivity-specificity ratio for END-IT, and high specificity for EMSE [7]. However, EEG data (used for EMSE outcome-predication) of the patients were excluded, and ENT-IT was not performed in every patient due to unavailable of brain image. Herein, we investigated the effectiveness of these scoring systems in the prediction of functional outcomes both at and after discharge in SE patients from our neurological intensive care unit (NICU).

## 2. Materials and Methods

### 2.1. Study Design

We reviewed and examined the medical records of all patients admitted for SE in the NICU at Kaohsiung Chang Gung Memorial Hospital between January 2017 and December 2017. This study was approved by the Chang Gung Medical Foundation Institutional Review Board (IRB No.: 201900016B0).

### 2.2. Definitions and Criteria

SE was defined as continuous clinical and/or electrographic seizure activity that lasted for at least five minutes, or recurrent seizure activity with no recovery period (returning to baseline) between seizures [8]. Clinical information was recorded using a standardized evaluation form. Acquired data included the age of the patients, duration of admission and last follow-up, type of SE treatment, and the outcomes at discharge and last follow-up. The follow-up phase of our study started at the time of discharge and terminated at our study endpoint (November 2018). The semiology and etiology of SE, as well as additional parameters, were recorded accordingly as defined in the different scoring systems. SE that did not respond to first-line (benzodiazepine) or second-line therapy and required therapeutic general anesthesia was defined as refractory SE [9]. SE that lasted 24 h or more after the initiation of anesthesia, including those that recurred upon the reduction or withdrawal of anesthesia, was defined as super-refractory SE [9].

STESS was determined using factors including age, history of seizure, the worst pre-treatment seizure type, and pre-treatment consciousness level [4]. These data were collected at the time of admission. The EMSE requires information about the age, etiology of SE, comorbidity, and the worse EEG pattern [5] and END-IT is scored by parameters regarding whether or not the worst SE is nonconvulsive, if encephalitis is the etiology, intubated or not, resistance to benzodiazepines or not, and the brain image study [6]. For EMSE and END-IT, as some of the considered parameters were subject to changes during the course of hospitalization, we determined two EMSE scores and two END-IT scores at two separate time points. Specifically, the first EMSE, designated EMSE^1^, was determined by analyzing the first available EEG study and the suspected etiology at the time of admission. The second and final EMSE, designated EMSE^f^, analyzed the worst EEG pattern obtained during the course of hospitalization and the confirmed etiology at discharge or upon death. For the END-IT method, the first END-IT score, designated END-IT^1^, analyzed information obtained at the time of admission. The second and final END-IT score, designated END-IT^f^, was determined using newly acquired information during the course of hospitalization. As previously recommended, cutoff values of ≥3, ≥64, and ≥3 were considered poor prognostic outcome predictions for STESS, EMSE, and END-IT, respectively [4,5,6]. To determine the accuracy of these prediction scores, clinical outcomes at discharge and at last follow-up were graded using the modified Rankin Scale (mRS). An mRS score of <3 was considered a good outcome and an mRS score of ≥3 was considered a poor outcome.

### 2.3. Statistical Analysis

Statistical analyses were performed using the IBM SPSS Statistics for Windows (version 22; IBM Corp., Armonk, NY, USA). A receiver operating characteristic (ROC) curve was generated for each scoring systems and the area under the curve (AUC) was compared using the method previously proposed by Hanley and McNeil [10]. Based on our designated cutoff values, the sensitivity, specificity, and correct classification rate were calculated for each score. McNemar’s test was used to compare the sensitivity, specificity, and correct classification rate between scores. A *p* value of less than 0.05 was considered to be statistically significant.

## 3. Results

### 3.1. Patients Cohort

The reviewed cohort consisted of 55 patients, including 23 females (41.8%) and 32 males (58.2%), and the median age at admission was 65 years (interquartile range = 56–79). Of these 55 patients, 16 (29.1%) were diagnosed with refractory SE and six patients (10.9%) were diagnosed with super-refractory SE. The median duration of admission was 15 days (interquartile range = 9–28), and nine patients (16.4%) died during admission. The median follow-up period post-discharge was 372 days (interquartile range = 122.8–455.3). The detailed demographic data are presented in Table 1. Thirty-nine patients (70.9%) had STESS ≥3, 16 (29.1%) had EMSE^1^ ≥64, 18 (32.7%) had EMSE^f^ ≥64, 10 (18.2%) had END-IT^1^ ≥3, and 11 (20%) had END-IT^f^ ≥3. The detailed score parameters for STESS, EMSE, and END-IT are presented in Table 2, Table 3 and Table 4, respectively. EMSE^1^ and EMSE^f^ were different in 16 patients (29.1%), with six showing improvement (EMSE^f^ < EMSE^1^) and 10 showing deterioration (EMSE^f^ > EMSE^1^). END-IT^1^ and END-IT^f^ were different in eight patients (14.5%), with two showing improvement (END-IT^f^ < END-IT^1^) and six patients showing deterioration (END-IT^f^ > END-IT^1^). Lastly, 15 patients (27.3%) and 16 patients (29.1%) had good outcome scores at discharge and during follow-up, respectively.

### 3.2. Comparisons of Different Outcome Prediction Scores at Discharge

**Figure 1:**
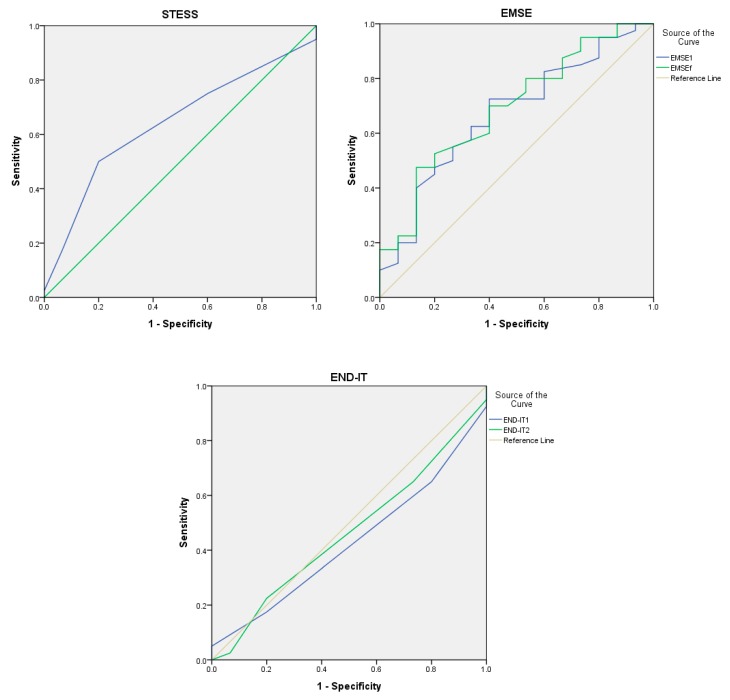
Receiver operating characteristic curve of STESS, EMSE, and END-IT for the prediction of the outcome at discharge. Abbreviations: EMSE = Epidemiology-Based Mortality Score in Status Epilepticus; END-IT = Encephalitis-Nonconvulsive Status Epilepticus-Diazepam Resistance-Image Abnormalities-Tracheal Intubation; STESS = Status Epilepticus Severity Score. EMSE1 and END-IT1 referred to the scores obtained at the time of admission and EMSEf and END-ITf referred to that obtained by reviewing the data during the course of hospitalization.

The ROC curves of the outcome prediction scores at discharge are presented in Figure 1. Our results suggested that AUC values were similar among STESS (0.629; 95% CI, 0.471–0.787), EMSE^1^ (0.669; 95% CI, 0.510–0.828), and EMSE^f^ (0.692; 95% CI, 0.537–0.846), while AUC values were below 0.5 for END-IT^1^ (0.428; 95% CI, 0.267–0.588) and END-IT^f^ (0.464; 95% CI, 0.298–0.631). Comparative analysis revealed no statistical significance (*p* >0.05) between AUC values.

**Figure 2:**
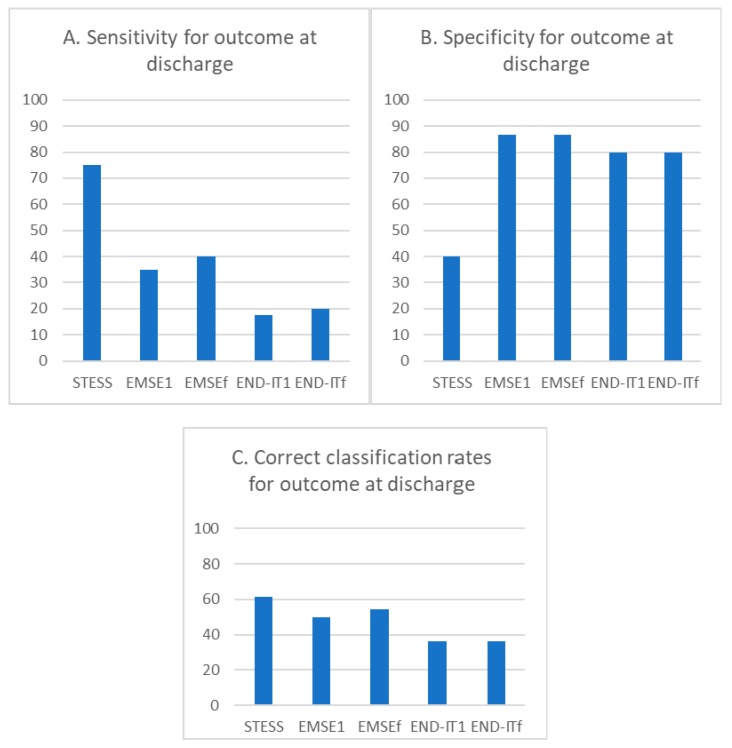
The bar chart of the percentage of sensitivity, specificity, and correct classification rates for STESS, EMSE, and END-IT at predicting the outcome at discharge. Abbreviations: EMSE = Epidemiology-Based Mortality Score in Status Epilepticus; END-IT = Encephalitis-Nonconvulsive Status Epilepticus-Diazepam Resistance-Image Abnormalities-Tracheal Intubation; STESS = Status Epilepticus Severity Score. EMSE1 and END-IT1 referred to the scores obtained at the time of admission and EMSEf and END-ITf referred to that obtained by reviewing the data during the course of hospitalization.

The sensitivity for poor outcome predictions at discharge for STESS (≥3), EMSE^1^ (≥64), EMSE^f^ (≥64), END-IT^1^ (≥3), and END-IT^f^ (≥3) were 75.0%, 35%, 40.0%, 17.5%, and 20.0%, respectively (Figure 2A). The specificity for poor outcome predictions at discharge for STESS (≥3), EMSE^1^ (≥64), EMSE^f^ (≥64), END-IT^1^ (≥3), and END-IT^f^ (≥3) were 40.0%, 86.7%, 86.7%, 80.0%, and 80.0%, respectively (Figure 2B). The correct classification rates for STESS, EMSE^1^, EMSE^f^, END-IT^1^, and END-IT^f^ were 61.4%, 50.0%, 54.5%, 36.4%, and 36.4%, respectively (Figure 2C).

The pairwise comparisons of all scores using McNemar’s test are shown in Table 5. STESS was significantly more sensitive than EMSE^1^, EMSE^f^ and END-IT^f^ (*p* = 0.02, 0.02, and 0.035, respectively) but less specific than all other scores (*p* < 0.05). Moreover, the END-IT^1^ exhibited lower specificity compared with EMSE^1^ and EMSE^f^ (*p* = 0.046 and 0.018, respectively). The END-IT^f^ exhibited significantly lower specificity compared with EMSE^f^ (*p* = 0.038). END-IT^1^ exhibited a significantly lower correct classification rate compared with STESS or EMSE^f^ (*p* = 0.031 and 0.038, respectively), and END-IT^f^ exhibited a significantly lower correct classification rate compared with STESS or EMSE^f^ (*p* = 0.026 and 0.038, respectively).

### 3.3. Comparison of Different Outcome Predictive Scores at Last Follow-up

**Figure 3:**
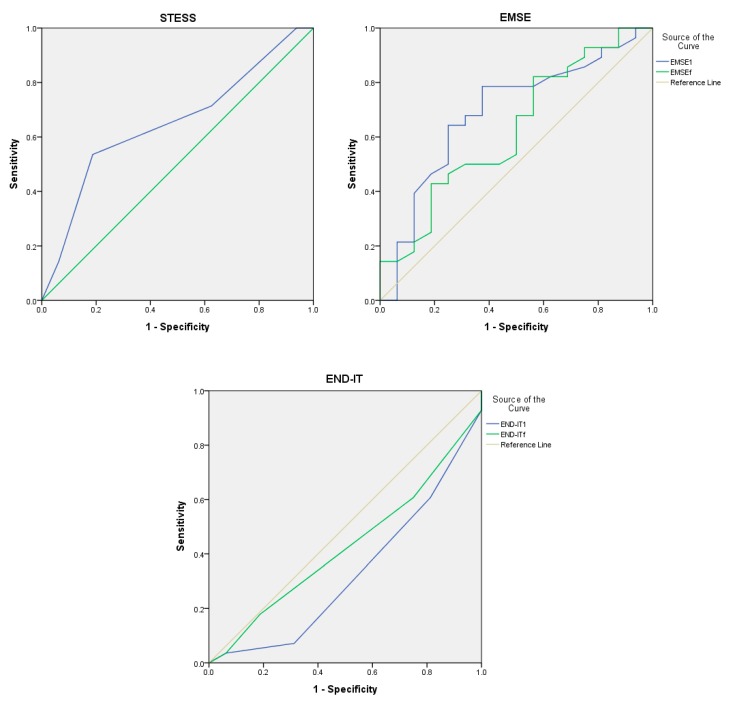
Receiver operating characteristic curve of STESS, EMSE, and END-IT for the prediction of the outcome at last follow up. Abbreviations: EMSE = Epidemiology-Based Mortality Score in Status Epilepticus; END-IT = Encephalitis-Nonconvulsive Status Epilepticus-Diazepam Resistance-Image Abnormalities- Tracheal Intubation; STESS = Status Epilepticus Severity Score. EMSE1 and END-IT1 referred to the scores obtained at the time of admission and EMSEf and END-ITf referred to that obtained by reviewing the data during the course of hospitalization.

Evaluations of the outcomes at last follow-up were performed in 44 patients. The ROC curves of the scoring systems for outcome predictions at follow-up are presented in Figure 3. The AUC values were similar for STESS (0.651; 95% CI, 0.484–0.817), EMSE^1^ (0.690; 95% CI, 0.522–0.857), and EMSE^f^ (0.626; 95% CI, 0.452–0.800), and were below 0.5 for END-IT^1^ (0.328; 95% CI, 0.161–0.495), and END-IT^f^ (0.427; 95% CI, 0.255–0.600). Comparative analysis revealed no statistical significance (*p* > 0.05) between the AUC values.

**Figure 4:**
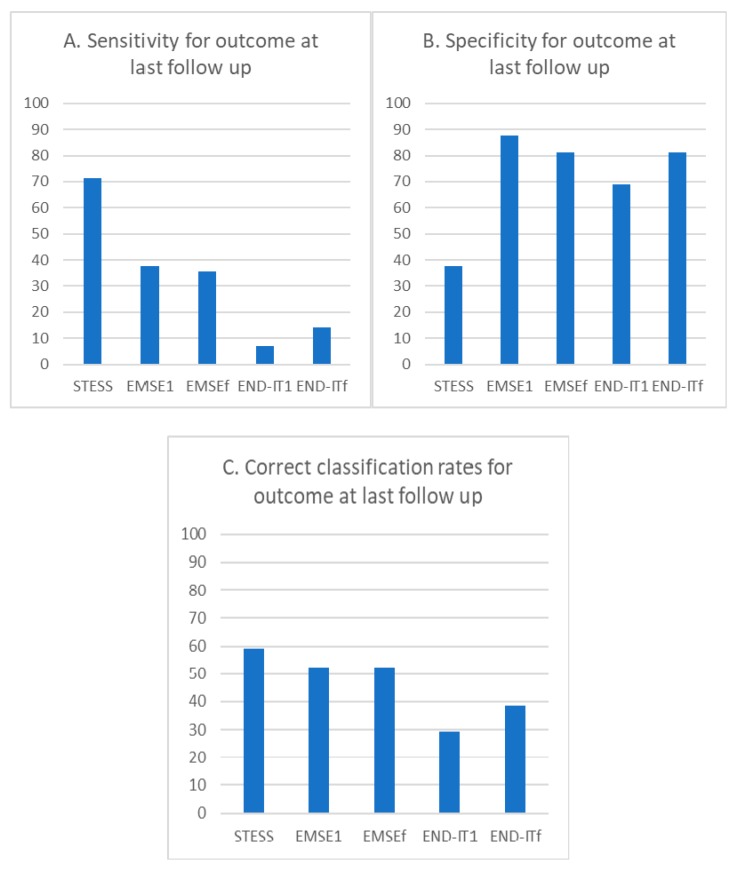
The bar chart of the percentage of sensitivity, specificity, and correct classification rates for STESS, EMSE, and END-IT at predicting the outcome at last follow up. Abbreviations: EMSE = Epidemiology-Based Mortality Score in Status Epilepticus; END-IT = Encephalitis-Nonconvulsive Status Epilepticus-Diazepam Resistance-Image Abnormalities- Tracheal Intubation; STESS = Status Epilepticus Severity Score. EMSE1 and END-IT1 referred to the scores obtained at the time of admission and EMSEf and END-ITf referred to that obtained by reviewing the data during the course of hospitalization.

The sensitivity for poor outcome predictions at last follow-up for STESS (≥3), EMSE^1^ (≥64), EMSE^f^ (≥64), END-IT^1^ (≥3), and END-IT^f^ (≥3) were 71.4%, 37.5%, 35.7%, 7.1%, and 14.3%, respectively (Figure 4A). The specificity for poor outcome predictions at last follow-up for STESS (≥3), EMSE^1^ (≥64), EMSE^f^ (≥64), END-IT^1^ (≥3), and END-IT^f^ (≥3) were 37.5%, 87.5%, 81.3%, 68.8%, and 81.3%, respectively (Figure 4B). The correct classification rates for STESS, EMSE^1^, EMSE^f^, END-IT^1^, and END-IT^f^ were 59.1%, 52.3%, 52.3%, 29.5%, and 38.6%, respectively (Figure 4C).

The pairwise comparisons of all scores using McNemar’s test are shown in Table 6. Our results indicated that STESS was significantly more sensitive than EMSE^1^, EMSE^f^, or END-IT^f^ (*p* = 0.011, 0.033, and 0.020, respectively), but significantly less specific than all other scores (*p* < 0.05). The END-IT^1^ exhibited lower specificity compared with EMSE^1^ or EMSE^f^ (*p* = 0.020 and 0.011, respectively). Similarly, END-IT^1^ exhibited a significantly lower correct classification rate compared with STESS, EMSE^1^, or EMSE^f^ (*p* = 0.012, 0.006, and 0.011, respectively).

## 4. Discussion

In this study, we examined the effectiveness of currently available scoring systems in the prediction of functional outcomes in SE patients at discharge and approximately one year post-discharge. Overall, our data indicated that STESS was the most sensitive predictive scoring method and EMSE was the most specific predictive scoring method for SE outcome predictions.

The initial presentation of SE patients does not usually provide all of the information necessary for EMSE and END-IT. Moreover, the clinical information of patients may change as the disease progresses, which would cause fluctuations in EMSE and END-IT scores. To control for these potential discrepancies, EMSE and END-IT were determined at two different time points. Our data nevertheless revealed no significant differences in sensitivity, specificity, or correct classification rate for outcome predictions between the EMSE and END-IT values obtained at different time points. These results suggested that EMSE and END-IT conducted at initial presentation with limited data (EMSE^1^ and END-IT^1^) were sufficiently accurate for the prediction of functional outcomes in SE patients.

The STESS and EMSE were originally developed to predict the in-hospital mortality of SE patients [4,5], whereas the END-IT was developed to predict the mRS three months after discharge [6]. In surviving patients, SE may lead to neurological and cognitive deficits and cause severe disabilities [11,12]. It is therefore important for neurologists to predict not only mortality but also functional outcomes when making treatment decisions. Our study revealed that EMSE^1^ had the highest AUC for outcome prediction both at discharge and post-discharge, while STESS and EMSE^1^ had the highest sensitivity and specificity in predicting poor outcomes, respectively. On the other hand, END-IT^1^ had the lowest AUC and the poorest correct classification rate. Previously, Kang et al. tested STESS and EMSE in predicting the functional outcomes in 120 patients at discharge and found that EMSE had a significantly higher AUC than STESS [13]. Albeit statistically insignificantly, similar trends were observed in our study. Similarly, Giovannini et al. analyzed 162 patients and compared mRS changes before admission and 30 days post-discharge and found that while EMSE was more specific than STESS, no difference in sensitivity was observed between both tests [14].

END-IT performed most poorly in our study cohort. This may partly be due to under-categorization of risk factor. For example, encephalitis is used in END-IT as a general indicator for poor outcomes. However, recent studies have suggested that infectious encephalitis and autoimmune encephalitis may have significantly different outcomes. Indeed, infectious encephalitis is associated with up to 71.4% mortality and poor neurological outcomes in 56% of patients [1]. Autoimmune encephalitis, on the other hand, generally predicts good outcomes in 45.6–72.6% of patients [15], if appropriate immunotherapy is prescribed accordingly [15,16]. Moreover, in the founding study of END-IT, 91.5% of the encephalitis group had infectious encephalitis [6]. However, autoimmune encephalitis had emerged as an important etiology of SE recently [17] and several studies including ours have shown that infectious and autoimmune encephalitis have similar prevalence among SE patients [18,19,20]. Thus, SE patients with autoimmune encephalitis may generally have better outcomes than predicated by END-IT. These results indicated that the END-IT method may not be representative as originally intended and may explain the poor predictive power as revealed in this study.

The AUC of STESS and EMSE were relatively low in our study, ranged between 0.6–0.7. Most previous studies from different geographic regions reported similar figures, ranged from 0.5–0.7 [7,13,14], except two studies reported AUC ranged between 0.7–0.89 for both tests [21,22]. The reason for lower AUC may be due to the samples size and different ethnicity studied in this cohort. Nonetheless, these studies give us a glance of how the scoring systems work in different populations. Although STESS and EMSE were good predictors for outcome analysis, the suboptimal AUC and the discrepancy among studies demands the development of a better scoring system.

Our study was limited by its retrospective approach that it only involved a single tertiary medical center in southern Taiwan. A larger patient cohort with increased geographic coverage may better evaluate the effectiveness and representativeness of these scores. Another pitfall of this study was that all these scores are subject to the population-dependent “classical test theory” [23]. Future studies involving populations of different ethnicity may clarify the universal applicability of these predictive scoring methods.

In conclusion, we found that while STESS was the most sensitive method for outcome predictions in SE patients, EMSE was the most specific method for outcome predictions in SE patients. We found that END-IT predicted functional outcomes in SE patients poorly, and that fluctuations in EMSE and END-IT scores did not cause any significant impacts on their powers of prediction. Finally, we confirmed that STESS and EMSE predictions can accurately reflect functional outcomes in SE patients both at the time of discharge as well as in the long term.

## Figures and Tables

**Table 1:** Demographic data of the studied population.

	Patients (%) N = 55
Age at admission (years)	65 (56–79) †
Female	23 (41.8)
STESS ≥3	39 (70.9)
EMSE^1^ * ≥64	16 (29.1)
EMSE^f^ * ≥64	18 (32.7)
END-IT^1^ * ≥3	10 (18.2)
END-IT^f^ * ≥3	11 (20.0)
Refractory SE	16 (29.1)
Super refractory SE	6 (10.9)
Days of admission	15 (9–28)
Death during admission	9 (16.4)
Duration of follow up (Days)	372 (122.8–455.3) †
Good outcome at discharge (mRS≤2)	15 (27.3)
Good outcome at last follow up (mRS≤2)	16 (29.1)

† Continuous variables were presented as median (interquartile range). Categorical variables were presented as N (%). Abbreviations: EMSE = Epidemiology-Based Mortality Score in Status Epilepticus; END-IT = Encephalitis-Nonconvulsive Status Epilepticus-Diazepam; Resistance-Image Abnormalities-Tracheal Intubation; mRS = modified Rankin Scale; SE = status epilepticus; STESS = Status Epilepticus Severity Score. * The superscript 1 in EMSE and END-IT referred to the scores obtained at the time of admission. The superscript f in EMSE and END-IT referred to the scores obtained by reviewing the data during the course of hospitalization.

**Table 2:** Distribution of the parameters in Status Epilepticus Severity Score.

Parameter	Value	N (%)
Level of consciousness before treatment	Alert, somnolent, or confused	8 (14.5)
	Stuporous or comatose	47 (85.5)
Worst seizure type before treatment	Focal motor, absence, or myoclonic seizure	13 (23.6)
	Generalized motor seizure	37 (67.3)
	Nonconvulsive status epilepticus	5 (9.1)
Age	<65 years	26 (47.3)
	≥65 years	29 (52.7)
History of previous seizures	Yes	24 (43.6)
	No or unknown	31 (56.4)

Categorical variables were presented as N (%).

**Table 3:** Distribution of the parameters in Epidemiology-Based Mortality Score in Status Epilepticus (EMSE).

Parameter	Value	EMSE^1^ *	EMSE^f^ *
Etiology	AED withdrawal or poor compliance	5 (9.1)	7 (12.7)
	Remote cerebrovascular disease	22 (40.0)	19 (34.5)
	Hydrocephalus	0 (0)	2 (3.6)
	Alcohol related	2 (3.6)	2 (3.6)
	Head trauma	3 (10.9)	2 (3.6)
	Unknown	3 (5.5)	4 (7.3)
	Brain tumor	3 (5.5)	3 (5.5)
	Sodium imbalance	4 (7.3)	4 (7.3)
	Metabolic disorders	4 (7.3)	4 (7.3)
	Acute cerebrovascular disease	5 (9.1)	5 (9.1)
	Acute central nervous system infection	4 (7.3)	3 (5.5)
Age	31–40	6 (10.9)	6 (10.9)
	41–50	3 (5.5)	3 (5.5)
	61–60	9 (16.4)	9 (16.4)
	61–70	14 (25.5)	14 (25.5)
	71–80	9 (16.4)	9 (16.4)
	>80	14 (25.5)	14 (25.5)
Comorbidity#	0 point	8 (14.5)	8 (14.5)
	10 points	40 (72.7)	40 (72.7)
	20 points	22 (40.0)	22 (40.0)
	30 points	2 (3.6)	2 (3.6)
EEG	No LPDs, GPDs, or ASIDs	40 (72.7)	36 (65.5)
	Either LPDs, GPDs, or ASIDs	15 (27.3)	19 (34.5)
	Spontaneous burst suppression	0 (0)	0 (0)

Categorical variables were presented as N (%). Abbreviations: AED = antiepileptic drug; ASID = after status ictal discharges; LPD = lateralized periodic discharges; GPD = generalized sharply and/or triphasic periodic potentials. * The superscript 1 refers to the scores obtained at the time of admission and the superscript f refers to those obtained after admission during the course of hospitalization. # In the comorbidity parameter, 10 points imply having one of the following: myocardial infarction, congestive heart failure, peripheral vascular disease, cerebrovascular disease, dementia, chronic pulmonary disease, connective tissue disease, ulcer disease, mild liver disease, or diabetes mellitus; 20 points imply having one of the following: hemiplegia, moderate or severe renal disease, diabetes mellitus with end organ damage, or any tumor including leukemia or lymphoma; 30 points imply having moderate or severe liver disease.

**Table 4:** Distribution of the parameters in Encephalitis-Nonconvulsive Status Epilepticus-Diazepam Resistance-Image Abnormalities-Tracheal Intubation (END-IT).

Parameter	Value	END-IT^1^ *	END-IT^f^ *
Encephalitis	No	51 (92.7)	50 (90.9)
	Yes	4 (7.3)	5 (9.1)
Non-convulsive status epilepticus	No	50 (90.9)	49 (89.1)
	Yes	5 (9.1)	6 (10.9)
Resistance to benzodiazepines	No	30 (54.5)	30 (54.5)
	Yes	25 (45.5)	25 (45.5)
Image study	No responsible lesion	15 (27.3)	14 (25.5)
	Unilateral lesion	33 (60.0)	35 (63.6)
	Bilateral lesions	7 (12.7)	6 (10.9)
Tracheal intubation	No	34 (61.8)	30 (54.5)
	Yes	21 (38.2)	25 (45.5)

Categorical variables were presented as N (%). * The superscript 1 referred to the scores obtained at the time of admission and the superscript f referred to that obtained by reviewing the data during the course of hospitalization.

**Table 5:** Pairwise comparisons of sensitivities, specificities, and correct classification rates of the different scores at predicting the modified Rankin Scale at discharge.

**Sensitivity (%)**				
**STESS (75.0%)**				
**0.020**	EMSE^1^ (35.0%)			
**0.020**	1.000	EMSE^f^ (40.0%)		
0.055	0.500	0.500	END-IT^1^ (17.5%)	
**0.035**	0.500	0.500	0.750	END-IT^f^ (20.0%)
**Specificity (%)**				
STESS (40.0%)				
**0.000428**	EMSE^1^ (86.7%)			
**0.002**	0.250	EMSE^f^ (86.7%)		
**0.000003**	**0.046**	**0.018**	END-IT^1^ (80.0%)	
**0.000005**	0.090	**0.038**	0.500	END-IT^f^ (80.0%)
**Correct Classification Rate (%)**				
STESS (61.4%)				
0.202	EMSE^1^ (50.0%)			
0.339	0.250	EMSE^f^ (54.5%)		
**0.031**	0.090	**0.038**	END-IT^1^ (36.4%)	
**0.026**	0.090	**0.038**	0.688	END-IT^f^ (36.4%)

Comparisons should be read from left to right. The value of sensitivities, specificities, and correct classification rates for each score was in the parentheses behind it. The respective *p* values from McNemar’s test of the column vs row scoring tools were presented in the table grid. Statistically significant values (*p* < 0.05) were expressed in bold. Abbreviations: EMSE = Epidemiology-Based Mortality Score in Status Epilepticus; END-IT = Encephalitis-Nonconvulsive Status Epilepticus-Diazepam Resistance-Image Abnormalities- Tracheal Intubation; STESS = Status Epilepticus Severity Score. * The superscript 1 in EMSE and END-IT referred to the scores obtained at the time of admission. The superscript f in EMSE and END-IT referred to the scores obtained by reviewing the data during the course of hospitalization.

**Table 6:** Pairwise comparisons of sensitivities, specificities, and correct classification rates of the different scores at predicting the modified Rankin Scale at last follow up.

**Sensitivity (%)**				
**STESS (71.4%)**				
**0.011**	EMSE^1^ (37.5%)			
**0.033**	0.500	EMSE^f^ (35.7%)		
0.090	0.188	0.344	END-IT^1^ (7.1%)	
**0.020**	0.500	0.656	0.250	END-IT^f^ (14.3%)
**Specificity (%)**				
STESS (37.5%)				
**0.002**	EMSE^1^ (87.5%)			
**0.004**	0.500	EMSE^f^ (81.3%)		
**0.00002**	**0.020**	**0.011**	END-IT^1^ (68.8%)	
**0.000072**	0.090	0.055	0.250	END-IT^f^ (81.3%)
**Correct classification rate (%)**				
STESS (59.1%)				
0.339	EMSE^1^ (52.3%)			
0.339	0.750	EMSE^f^ (52.3%)		
**0.012**	**0.006**	**0.011**	END-IT^1^ (29.5%)	
0.061	0.090	0.105	0.063	END-IT^f^ (38.6%)

Comparisons should be read from left to right. The value of sensitivities, specificities, and correct classification rates for each score was in the parentheses behind it. The respective *p* values from McNemar’s test of the column vs row scoring tools were presented in the table grid. Statistically significant values (*p* < 0.05) were expressed in bold. Abbreviations: EMSE = Epidemiology-Based Mortality Score in Status Epilepticus; END-IT = Encephalitis-Nonconvulsive Status Epilepticus-Diazepam Resistance-Image Abnormalities- Tracheal Intubation; STESS = Status Epilepticus Severity Score. * The superscript 1 in EMSE and END-IT referred to the scores obtained at the time of admission. The superscript f in EMSE and END-IT referred to the scores obtained by reviewing the data during the course of hospitalization.
